# The defective RNAs of Closteroviridae

**DOI:** 10.3389/fmicb.2013.00132

**Published:** 2013-05-23

**Authors:** Moshe Bar-Joseph, Munir Mawassi

**Affiliations:** The S. Tolkowsky Laboratory, Virology Department, Plant Protection Institute, Agricultural Research OrganizationBeit Dagan, Israel

**Keywords:** citrus viruses, RNA viruses, RNA recombination, viral replicase, template-switching, non-replicative RNAs, virus replication, defective RNA

## Abstract

The family Closteroviridae consists of two genera, *Closterovirus* and *Ampelovirus* with monopartite genomes transmitted respectively by aphids and mealybugs and the Crinivirus with bipartite genomes transmitted by whiteflies. The Closteroviridae consists of more than 30 virus species, which differ considerably in their phytopathological significance. Some, like *beet yellows virus* and *citrus tristeza virus* (CTV) were associated for many decades with their respective hosts, sugar beets and citrus. Others, like the grapevine leafroll-associated ampeloviruses 1, and 3 were also associated with their grapevine hosts for long periods; however, difficulties in virus isolation hampered their molecular characterization. The majority of the recently identified Closteroviridae were probably associated with their vegetative propagated host plants for long periods and only detected through the considerable advances in dsRNA isolation and sequencing of PCR amplified replicons. Molecular characterization of CTV and several other Closteroviridae revealed that, in addition to genomic and subgenomic RNAs, infected plants contain several different subviral defective RNAs (dRNAs). The roles and biological functions of dRNAs associated with Closteroviridae remain terra incognita.

## LARGE RNA GENOMES AMONG THE CLOSTEROVIRIDAE: MEETING THE CHALLENGE OF SURVIVAL

Viruses are among the smallest biological entities and, because of the small size of their genomes, their survival depends on the use of a variety of molecular strategies that allow them to stretch their genome-coding capacities to the limit. For example, the single-stranded DNA Geminiviridae that are ~3 kb in length, a length that would generally allow transcription and translation of a less than 100-kDa polypeptide, employ a sophisticated multiframe reading of their plus-genome and negative-genome strands that amplifies their coding capacity several-folds, considerably increasing the number and size of the translation products that can be obtained (Hull, 2002). Similarly, the coding capacities of many single-stranded RNA viruses are enhanced by the use of read-through products. An even more common strategy is for a few viral expression products to play multiple roles. For example, the p25 gene of the *citrus tristeza closterovirus*, family Closteroviridae, (i) encodes the coat protein that encapsidates most (~97%) of the viral particles (Febres et al., 1996), (ii) acts as a suppressor of RNA silencing (RSS; Lu et al., 2004), and (iii) is most probably involved in vector adaptation for the natural transmission of this virus from infected to uninfected hosts. Similarly, the p23 protein of CTV serves as (i) an RSS (Lu et al., 2004), (ii) a regulator of RNA strand synthesis (Satyanarayana et al., 2002), and (iii) the inducer of CTV symptoms in certain CTV-sensitive host plants (Flores et al., 2013).

Viral genomes are equipped with a complex and versatile toolbox that allows them to survive and spread, despite a number of serious limitations, such as (i) dependence on just a few insect species for natural transmission, (ii) dependence on a sometimes restricted range of host plants or on only certain types of tissues, and (iii) the challenge of a potent generic host defense mechanism, the RNA-silencing system. Despite the sophisticated replication machinery that viruses have developed for their survival, it is difficult to understand how some large, single-stranded, single-component RNA genomes, such as the ~30-kb genome of animal-infecting coronaviruses and most of the 15- to 20-kb genomes of Closteroviridae members, manage to survive in the face of incidental degradation and targeted dicing by the active host defense silencing machinery within their host cells and/or as they are carried by phloem-feeding aphid vectors. The biological cost of such a situation, in which every single fracture at each of the c. 20,000 possible fragile targets could lead to RNA disruption and to total genetic and energetic loss, could have been detrimental to the continuous survival of such big genomes. In addition, CTV must also contend with the grave consequences of high error rates of viral RNA replication (Lauring and Andino, 2010), which for the large CTV genome is expected to result in average of at least two nucleotide changes per each genome/generation. For CTV tolerant *Citrus* sp. trees, which often survive tens and even up to 100 years, such a mutation rate could had been expected to result in a considerable genetic diversity. Surprisingly, however, analyses of CTV strains from spatially and temporally separated citrus trees revealed a highly conserved genome (Albiach-Martí et al., 2000). Further evidence for the remarkable genetic stability of closterovirus genomes (Dolja and Koonin, 2013), especially of the CTV, is the CTV-based vectors infecting citrus plants. Some of the CTV-based vectors infected citrus trees continued to express the inserted green fluorescent protein (GFP) gene for up to 7 years (Dawson, 2011). The genetic stability of CTV holds apparently only when plants are infected by a monotypic closterovirus isolate (Weng et al., 2007). Genetic analysis of a Floridian CTV isolate from citrus tree infected by three major CTV genotypes revealed numerous variants generated by promiscuous recombination between the major genotypes and additional divergence further increased genotypic complexity of the initial recombinants (Weng et al., 2007; Zhongguo Xiong, personal communication). These results raise the possibility of an unknown mechanism to limit accumulation of point mutation CTV mutants while preserving those generated through recombination. The presence of multiple defective dRNAs in trees infected with many CTV isolates further supports this possibility (Batuman et al., 2010).

## DEFECTIVE AND OTHER SUBVIRAL RNAs

In addition to genomic and subgenomic RNAs, virus-infected hosts often contain two different types of subviral RNAs: (i) satellite RNAs, with sequences that are mostly or completely unrelated to their “helper” viruses and (ii) dRNAs that do not interfere with their helpers, or defective interfering RNAs (DI-RNAs) whose presence results in less virus accumulation and often in milder symptoms. Interestingly, however, the DI-RNA of *turnip crinkle virus* (TCV) that reduces virus accumulation causes increased rather than decreased severity of disease symptoms (Li et al., 1989; White and Morris, 1995). These different types of virus-associated molecules are distinguished from viral genomic RNA by the fact that they are not required for normal virus propagation.

Only the replication of satellite RNAs and some dRNAs and DI-RNAs is dependent upon enzymes encoded by their helper viruses. However, a few other dRNAs are able to replicate autonomously in inoculated cells, although their ability to spread and move is restricted in intact plants. DI-RNAs and dRNAs have been reported for many animal and plant viruses and the characterization of their sequences has revealed a mosaic of truncated forms, suggesting a variety of situations that could have lead to their emergence. All dRNAs possess some of the *cis*-acting elements necessary for replication of the parent virus and all are missing some of the genetic elements necessary for some essential virus functions, such as replication, encapsidation, or the ability to spread within a host.

## A SHORT INTRODUCTION TO CTV AND THE CLOSTEROVIRIDAE

The Closteroviridae family includes viruses that have been known for at least seven to eight decades, such as *beet yellows virus* (BYV; Duffus, 1973) and CTV (Bar-Joseph et al., 1989). Other viruses of this family, particularly among those belonging to the *Crinivirus* genus, appear to have emerged more recently. Low virus titer and association with deciduous fruit tree and grapevine (*Vitis vinifera*) woody hosts that are polyphenol rich handicapped purification of many of these viruses and delayed their characterization until finding that Closteroviridae infections are associated with large amounts of dsRNA (Dodds and Bar-Joseph, 1983). The considerable progress achieved toward elucidation of the genomic functions of Closteroviridae has been widely reviewed in different journals and book chapters

Members of the genus *Closterovirus* are mainly transmitted by aphids; whereas members of the genus *Ampelovirus*, which also have monopartite genomes, are mainly transmitted by mealybugs (Martelli et al., 2012). The *Crinivirus*, and a recently proposed new genus with monopartite particles named *Velarivirus* (Al Rwahnih et al., 2012) are mostly transmitted by whiteflies. Most of the members of the genus *Crinivirus* (transmitted by whiteflies) have bipartite genomes, although a virus with a tripartite genome, *potato yellow vein virus* (PYVV), was assigned to this genus (Livieratos et al., 2004). Interestingly, the virus–vector relationships of members of the three Closteroviridae genera appear to have had far stronger effects on the diversification of a range of genomic properties than on the adaptation of these viruses to different hosts (Karasev, 2000).

**FIGURE 1:**
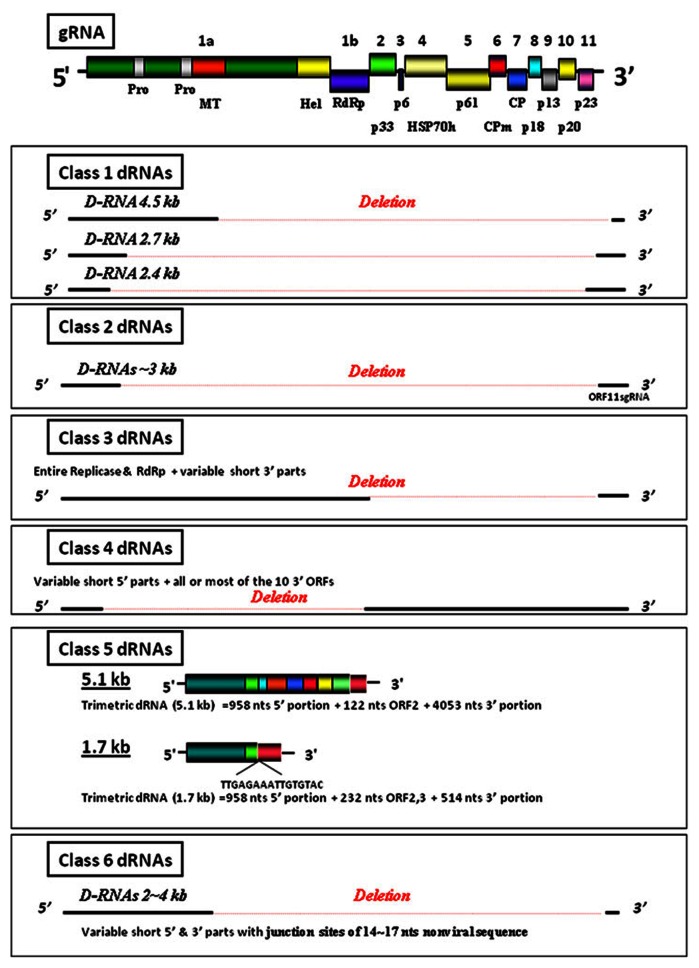
**A diagram of the *Citrus tristeza virus *(CTV) genomic RNA (gRNA).** The 5′ and 3′-termini of the genome, the ORFs with respective numbers and encoded proteins and protein domains are indicated. Pro, papain-like protease; MT, methyltransferase; Hel, RNA helicase; RdRp, RNA-dependent RNA polymerase; HSP70h, a heat shock protein 70 homolog; CP, major coat protein; CPm, minor coat protein. Proteins are named according to their relative molecular masses. The figure shows also diagrams of Classes 1–6 defective (D) RNAs associated with CTV gRNA.

A schematic presentation of the CTV genomic RNA is shown in **Figure 1**. Among the interesting features of this virus are the arrangement of the genome and the high level of similarity among the sizes of the full-length genomes of the CTV strains that have been characterized to date. The sequences of the 3′-halves of most strains of CTV are very similar. However, the 5′ end of the T36 strain differs from that of the VT and most of the other strains to the extent that, at a certain stage, virologists considered referring to these strains as two different species. In addition to the genomic RNA, which is also found in isolated virus particles, CTV-infected cells contain RF (replicative-form) RNA molecules that consist of full-length plus and minus strands and a large number (up to 30 or more) of 5′- and 3′-sgRNAs. Interestingly, while the 5′-sgRNAs are all single-stranded and positive, the 3′-sgRNAs include both positive- and minus-strand molecules. Readers are referred to several other chapters in this series, which provide considerably more detailed discussions of the CTV genome and the replication strategies of this virus.

## CLASSES OF CTV-dRNAs

One of the most prominent features of CTV, which was revealed soon after its genome was first characterized (Karasev et al., 1995), is that dRNA molecules are present in most isolates of this virus (Mawassi et al., 1995a, b; and for a recent review, see Batuman et al., 2010). Interestingly, T36, the first CTV strain to have its genome characterized, differs in this aspect. Unlike VT, in which almost every subtype contains major dRNAs, dRNAs appear to be less common in the T36 strain. This difference was probably responsible for one of the main bottlenecks in the early attempts to obtain a full-length genomic sequence of CTV-VT, despite partial cloning and efficient utilization of cDNA clones to distinguish between CTV strains (Rosner and Bar-Joseph, 1984).

Many CTV isolates contain one or more dRNAs of various sizes. Most of these dRNA molecules consist of two genomic termini, with extensive internal deletions (Mawassi et al., 1995a, b; Yang et al., 1997; Ayllon et al., 1999). It is interesting to note that only certain dRNAs are regularly transferred by mechanical transmission (Che et al., 2003) and most are not passed along during aphid transmission (P. Moreno, personal communication).

For convenience, CTV-defective RNAs (dRNAs) can be grouped into different classes. These classes are described below (see **Figure 1**).

### CLASS 1: CTV-dRNAs

Class 1 CTV-dRNAs possess different-sized 5′ and 3′ sequences that are not significantly homologous with one another (Mawassi et al., 1995a, b) and are thought to be the result of erroneous replication involving non-homologous recombination. The junction sites of the 2.3- and 4.5-kb dRNAs from CTV-VT (Mawassi et al., 1995b) are not flanked by direct repeats in the genomic RNA. Other dRNAs (Ayllon et al., 1999) contain direct repeats of 4–5 nt near their junction sites, supporting the possibility that they were generated via a replicase-driven template-switching mechanism.

### CLASS 2: CTV-dRNAs

Class 2 CTV-dRNAs possess 3′ moieties that are similar in size and structure to the full-length sgRNA of ORF11 (Yang et al., 1997). The extra cytosine at the junction sites of several of the dRNAs of this class corresponds to the extra guanine reported on the 3′ ends of minus strands of sgRNA and RF molecules (Karasev et al., 1997). These characteristics indicate that: (i) CTV-dRNA synthesis may have taken place through a process of template-switching of 5′ plus-strand molecules toward distal positions after the completion of minus-strand ORF11 sgRNA transcription and (ii) the 5′ of the ORF11 sgRNA might serve as a highly specific hotspot for RNA recombination (Bar-Joseph et al., 1997; Yang et al., 1997).

### CLASS 3: CTV-dRNAs

Class 3 CTV-dRNAs possess large (~12 kb) encapsidated dRNA molecules that are infectious when mechanically transmitted to citrus plants and *Nicotiana benthamiana* protoplasts. Their 5′ termini are identical to or slightly larger than the 5′ large, single-stranded sgRNA of ORF1a + 1b (LaMT) that have been reported in CTV-infected plants (Gowda et al., 2001; Che et al., 2002). The 3′ moiety of these large dRNAs varies and some have a truncated ORF11 (Che et al., 2002). Two of these junction sites start at the first nucleotide of the ORF10–11 intergenic region and one other coincides with the full-length sgRNA that codes for ORF10.

Artificially constructed large dRNAs that have intact ORF1a + b reading frames, but lack the translation products of all of the 3′ ORFs were found to self-replicate in protoplasts (Satyanarayana et al., 1999). Although these artificially constructed, defective molecules have not been found *in planta* and there is no evidence for their ability to spread systemically within inoculated plants, they have become useful genetic platforms for the study of sgRNA transcription (Gowda et al., 2001). The presence of insertions, deletions and inversions of 3′ sequences, including ORFs and their intergenic regions, in this self-replicating construct, allowed Gowda et al. (2001) to demonstrate that the production of a 5′-terminal positive strand and 3′-terminal positive- and negative-stranded sgRNA is permitted by each of the 3′ CTV controller elements.

### CLASS 4: CTV-dRNAs

Class 4: CTV-dRNAs are large, dRNAs, which retain all or most of the ten 3′ ORFs and appear to be analogous to the genomic RNA 2 of criniviruses. These large, dRNA molecules (LD-RNA2) can be transmitted to citrus plants by mechanical inoculation. However, the transmission of LD-RNA2 to protoplasts has been shown to be limited and cannot be detected by RT-PCR until 4 days after inoculation (Che et al., 2002, 2003).

### CLASS 5: CTV-dRNAs

Class 5: CTV-dRNAs vary in size (1.7–5.1 kb) and contain sequences that point to double-recombination events (DR). These sequences are comprised of two termini and a non-contiguous internal sequence from ORF2. Interestingly, LD-RNA2 and DR-dRNAs from three different CTV isolates all contain an identical 948-nt 5′ region.

### CLASS 6: CTV-dRNAs

Class 6: CTV-dRNAs have variable regions between the 5′ and 3′ termini and inserts of short (14–17 nt) sequences that have no homology with the CTV genome (Mawassi et al., 1995a, b; Yang et al., 1997; Che et al., 2003). The question of why these heterologous double-recombinants are so small has been raised. CTV-dRNA homologous-sequence double-recombinants (Class 5 dRNAs) are more than 100 nucleotides in length; whereas the heterologous inserts of Class 6 dRNAs are at least 10-fold shorter. One possible explanation for this could be that a naturally occurring selection process eliminates any CTV-dRNAs with inserts of host genes of 21–25 nt, which might silence the normal expression of the respective host genes. Such a mechanism could also explain the limited amount of non-self recombinants among RNA viruses, in general, and suggest a new function for RNA silencing of RNA viruses, namely reducing the possibility that virus genomes might amplify RNA segments derived from the mRNA of their hosts.

## DEFECTIVE RNAs ASSOCIATED WITH OTHER CLOSTEROVIRIDAE

Compared with CTV, the presence of dRNA in other viruses in this family has received far less attention. The few reports that have been published in this area include occasional observations of dRNA associated with the criniviruses *lettuce infectious yellows virus* (LIYV; Rubio et al., 2000), *lettuce chlorosis virus* (LCV; Mongkolsiriwattana et al., 2011), and PYVV (Eliasco et al., 2006) and the closterovirus *carrot yellow leaf virus* (CYLV; Menzel et al., 2009; W. Menzel, personal communication). In addition, a rather large dRNA (~6.0 kb) was reported in pineapple plants infected with the ampelovirus *pineapple mealybug wilt-associated virus-1* (PMWaV-1; Melzer et al., 2008). Thus, the presence of dRNA is a common feature of all three genera of the Closteroviridae family.

## DISCUSSION: STILL MORE QUESTIONS THAN ANSWERS

RNA recombination is the key process in the formation of the dRNA molecules associated with most animal and many plant viruses (White and Morris, 1995; Simon, 1999). Three different events have been suggested to lead to the recombination of viral RNA: breakage and ligation of incomplete RNA molecules, replicase-driven template-switching and breakage-induced template-switching (Nagy and Simon, 1997). Information gathered from Class 2 and 3 CTV-dRNAs led researchers to suggest a fourth mechanism involving the recombination of the 3′ termini of sgRNAs with different-sized pieces from the 5′ end of the CTV genome (Yang et al., 1997; Che et al., 2003). More recent studies have reported recombination involving the 5′ termini of sgRNAs and different-sized molecules from the 5′ part of the virus (see **Figure 1**, Classes 2 and 4). Components of the replication-associated machinery were found to be involved in the evolution of other viruses and intergenic regions are known to be the preferred crossover sites for brome mosaic virus (BMV) recombinants (Nagy and Bujarski, 1996).

The dRNAs of CTV provide us with some tentative answers to questions about virus evolution. First, the finding that populations of naturally self-replicating dRNAs (Class 3) and Class 4 dRNAs harboring the entire battery of ORFs analogous to the RNA 2 of criniviruses suggests that similar forms of dRNA may have led to the evolution of the bipartite criniviruses from a monopartite velarivirus parent. In addition, dsRNA molecules corresponding to one or more major CTV-dRNAs have been found to account for substantial amounts of the total dsRNA found in CTV-infected plants. Since dsRNA molecules can be considered the dead ends of the viral replication process, their abundance in Closteroviridae (Dodds and Bar-Joseph, 1983) naturally raises evolutionary questions. One possibility that we would like to suggest is that, in addition to the three suppressor genes, CTV and other Closteroviridae with large, very fragile genomes use the abundant dsRNA in their genomic, subgenomic, and dRNA as a buffering system, to protect their large RNA genomes against the risk of being targeted by the active host defense RNA silencing.

In our early work, we could not associate any specific biological phenomena with the 2.4-kb dRNA or some of the other dRNA molecules. After two decades of continuous transmission of the VT isolate in Mexican lime and Alemow seedlings, the symptoms observed on the Alemow plants remained unchanged. However, among the SO (sour orange) plants that used to test for SY (seedling yellows), we noticed that some failed to exhibit the typical SY symptoms. The examination of the dRNAs of Alemow plants infected with CTV-VT subisolates that exhibited SY and non-SY reactions revealed the presence of one major dRNA population. The main difference between both subisolate groups was the association of 4.5- and 2.4-kb dRNAs with non-SY- and SY-reacting VT isolates, respectively. Furthermore, VT isolate #12, which contains a large dRNA with a complete 5′ moiety of the gRNA, did not induce the stem-pitting symptoms typically observed in infected Alemow plants. However, the possibility of differences between SY and non-SY, or stem pitting(SP) and non-SP, isolates in other genomic regions was not ruled out and the considerable progress that has been made recently in the field of sequencing techniques is expected to clarify this issue. In conclusion, the roles and biological functions of the numerous dRNAs associated with Closteroviridae remain *terra incognita*.

## Conflict of Interest Statement

The authors declare that the research was conducted in the absence of any commercial or financial relationships that could be construed as a potential conflict of interest.
